# Microenvironment meets lineage complexity in junctional tumorigenesis

**DOI:** 10.1038/s41467-019-11651-6

**Published:** 2019-08-23

**Authors:** Wa Xian, Frank McKeon

**Affiliations:** 1 Institute of Molecular Medicine, McGovern Medical School of The University of Texas Health Science Center, Houston, TX 77030 USA; 2 Department of Biochemistry and Molecular Biology, University of Texas McGovern Medical School, Houston, TX 77030 USA; 3 Department of Biology and Biochemistry, University of Houston, Houston, TX 77204 USA

**Keywords:** Gastric cancer, Cancer microenvironment, Oesophageal cancer

## Abstract

Using a sensitizing genetic model, Moon and colleagues provide compelling data for a determinant role of microenvironment in tumorigenesis, and lend support to the notion that such influences can be pharmacologically dampened to reduce the onset of cancers.

Tumors arising at the gastroesophageal junction (“junctional tumors”) include esophageal adenocarcinoma (EAC), with or without the Barrett’s esophagus precursor lesion, gastric adenocarcinomas (GAC), including intestinal GAC which is similar in many regards to EAC as well as hereditary, diffuse gastric cancer (HDGC), and esophageal squamous cell cancer (ESCC), which is much like those appearing elsewhere along the esophagus^1^. Unraveling this panoply of cancers at the junction has been challenging from both the pathology and molecular standpoints and yet is critical for providing the most appropriate therapies and for devising preemptive approaches to target precursor lesions. The recent work by Moon et al.^2^ is thus timely in that it demonstrates, using a sensitized genetic model for the evolution of ESCC, why the location-dependent microenvironment along the esophageal axis and especially at the junction can be determinant for tumorigenesis. Their work also raises the possibility that not all esophageal stem cells along this axis are equal either due to their respective local environments, subtle differences in their epigenetic makeup, or some amalgam of the two. Finally, Moon et al. show through pharmacological modifications of the microenvironment or signaling pathways of sensitized cells that they can impact tumorigenesis. This latter finding may help to foster efforts parallel to the preemptive strategies currently under exploration^3–5^ to counter the recent rise in EAC incidence^6^.

## Fitting into the junction

Moon et al.’s findings add to the notoriety of the gastroesophageal junction previously implicated at the source of Barrett’s esophagus, a relatively common, precancerous lesion in the distal esophagus that greatly enhances the risk for EAC^1,6^. The fact that Barrett’s is an “intestinal metaplasia” provided no obvious path to its ontogeny from a junction thought to be a simple merging of esophageal squamous and gastric columnar epithelia. This conundrum set off a decades-long quest to define the origin of Barrett’s that yielded multiple and mutually exclusive theories evoking “transcommitment” of either esophageal or gastric stem cells to an intestinal metaplasia fate (reviewed in ref. ^7^). The underlying data supporting these contentions have always been fragile, but somehow the esophageal transcommittment hypothesis came to dominate accepted opinion. This is despite the fact that it ran counter to uniform clinical observations that Barrett’s in the distal esophagus is always continuous with the junction rather than sporadically distributed along the axis of the esophagus as would be predicted by the esophageal transcommitment hypothesis^7^. Our own work in this area was predicated on a mouse model that lacked the esophageal lineage altogether and yet ironically displayed an intestinal metaplasia with gene expression profiles highly similar to human Barrett’s^8^. This finding on its face precluded the esophageal transcommittment hypothesis and triggered a more detailed developmental biology analysis for the source of Barrett’s esophagus. It was clear from this analysis that the respective lineages that give rise to esophageal, gastric, and the intervening Barrett’s stem cells of the mature junction were already well-established and distinct by midgestation of murine embryogenesis^8^. This study also revealed that Barrett’s emerges from a previously unrecognized and preexisting population of cells that reside precisely at the interface between the esophageal and gastric cells in all normal mice and humans. The preexistence of these junctional cells explains why Barrett’s can emerge so rapidly, in a matter of days, upon genetically induced damage to the murine esophagus. The speed by which Barrett’s can expand into damaged esophagus also predicted that at least some clinically-defined Barrett’s would be devoid of mutations altogether. These notions were put to the test in a first-of-its-kind cloning of patient-matched stem cells of Barrett’s esophagus, gastric epithelia, and esophageal epithelia from 12 Barrett’s cases^9^. As might be expected, most of the Barrett’s stem cells had acquired multiple somatic mutations impacting p16, ARIDA1, and other genes seen for Barrett’s esophagus, but a third of the Barrett’s cases were, like their normal esophageal and gastric counterparts, devoid of somatic mutations. Lastly, the Barrett’s stem cells and their in vitro-differentiated epithelia share many of the molecular markers of the pre-existing, quiescent Barrett’s cells at the normal gastroesophageal junction in mice and humans^8,9^.

More recently, this notion of a junction involving three lineages in a polarized axis (ESO-BE-GAST) has been modified somewhat by the discovery of a non-squamous, stratifying epithelial population at the junction itself^10^. Like the rest of the esophagus, this non-squamous population expresses Krt5, p63, and Krt15, and seems to occupy the junction immediately anterior to the pre-existing Barrett’s glands in the junction. However, Jiang et al.^10^ argued that this non-squamous, p63+ cell population is also the source of Barrett’s esophagus. Their evidence was based on in vitro studies that these non-squamous epithelia cells have the potential to form columnar intestinal metaplasia upon expression of exogenous Cdx2, though the histology of their Cdx2-expressing cells appeared to retain a stratified appearance. Jiang et al. provided an array of “lineage-tracing” experiments that were said to support the concept that these p63+ non-squamous epithelia are the source of Barrett’s in the p63-null mouse.

The findings by Moon et al.^2^ therefore are very timely with regards to this newly recognized subset of esophageal epithelia at the junction and for junction tumorigenesis in general. For one, they show in their sensitized model of ESCC tumorigenesis that the Krt5, Krt15, p63+ positive cells adjacent to the junction are especially prone to progressing to papillomas compared to more anterior regions of the esophagus harboring the identical combination of activated oncogenes and tumor suppressor mutations. Moreover, these junctional papillomas continue to express Krt6, which is both a marker of migrating p63+ epithelia in response to wound healing in the skin^11^ and lung^12^ and a known marker of the most distal extension of the esophageal epithelium at the junction^8^. In light of the Moon et al. data, and the relative distribution of the Krt6+, p63+ cells immediately anterior to the Barrett’s progenitors at the junction, it is likely that the Krt6+, p63+ cells play some dynamic role to corralling the Barrett’s progenitors from migrating into the esophagus. Regardless, even with the induction of activated oncogenes and loss of p53, these junctional p63+/Krt5+ cells steadfastly retain their esophageal fate and do not undergo “transcommitment” to Barrett’s.

## The sensitizing model for ESCC

ESCC tumors are thought to arise from esophageal stem cell lineages (p63+, Krt5+, and Krt15+) and remain difficult to manage with a median survival of less than two years^1^. Detailed molecular genetics and epigenetics of ESCC indicate that these tumors fall into at least three mutational “types” underscoring the major challenges ahead for addressing advanced cancers of this sort^13,14^. Elegant studies dissecting the molecular evolution of ESCC are setting the stage for preemptive strategies aimed at key transitions along the path from precancerous lesions, dysplasia, and ESCC^15^. Part and parcel to these efforts are understanding the risk factors for ESCC, which again are proving to be more complex than anticipated^1^. For instance, smoking and alcohol consumption are established risk factors for ESCC in the West, while these associations are less obvious in Chinese cases of ESCC, which account for 50% of all cases in the world^1^. Given these complexities, Moon et al. asked whether esophageal stem cells sensitized for tumorigenesis via conditional expression of oncogenes and loss of tumor suppressors could reveal differential risk for progression as a function of their anterior-posterior location within the esophagus. They found that although they could genetically induce activated Ras expression and p53 loss across the Krt5+ stem cells of the esophageal axis, papilloma formation was heavily biased for the squamocolumnar junction. Hypothesising that this propensity for tumorigenesis at the junction was a reflection of proximity to acid producing cells of the murine hind stomach, Moon et al. tested the proton pump inhibitor (PPI) Pantoprazole and showed that it suppressed tumorigenesis and the associated inflammation in their model. They further explored the possibility that the low pH induction of tumorigenesis was operating through inflammatory pathways involving Cox-2, a pro-inflammatory mediator known to be expressed in ESCCs. They showed that the Cox-2 inhibitor celecoxib reduced the hypercellularity of organoids derived from esophageal progenitors expressing activated Ras in a p53-deficient background, and that the genetic loss of Cox2 in their murine model significantly suppressed tumorigenesis across the esophagus. Taken together, the data of Moon et al. raise the possibility of pharmacological suppression of either the initiation or progression of mutationally sensitized cells toward ESCC or at least a subset of ESCCs emerging near the gastroesophageal junction. As such, Moon et al. is an allusion to potentially exciting directions in EAC that suggest that statins, NSAIDs, and acid suppression may reduce the development of Barrett’s and/or its progression to EAC^3–5^. Coupled with reducing lifestyle risks via weight loss and smoking cessation, one or more of these medications could counter the enormous rise of EAC cases in developed countries over the past several decades. Statins in particular may be particularly effective due to their wide use and association with both lower rates of Barrett’s formation in at-risk patients and remarkable reductions in the progression of Barrett’s to EAC^4,5^.

Finally, Moon et al. underscores an emerging sense of the multiple lineages that comprise the gastroesophageal junction, their contributions to precancerous lesions for distal esophageal and junctional tumors, and the potential of pharmacological interventions to reduce the onset of lethal disease.
